# 2-(5-Fluoro-3-isopropyl­sulfanyl-1-benzofuran-2-yl)acetic acid

**DOI:** 10.1107/S1600536811044436

**Published:** 2011-10-29

**Authors:** Pil Ja Seo, Hong Dae Choi, Byeng Wha Son, Uk Lee

**Affiliations:** a Department of Chemistry, Dongeui University, San 24 Kaya-dong Busanjin-gu, Busan 614-714, Republic of Korea; b Department of Chemistry, Pukyong National University, 599-1 Daeyeon 3-dong, Nam-gu, Busan 608-737, Republic of Korea

## Abstract

The title compound, C_13_H_13_FO_3_S, was prepared by alkaline hydrolysis of ethyl 2-(5-fluoro-3-isopropyl­sulfanyl-1-benzofuran-2-yl)acetate. In the crystal, the carb­oxy groups are involved in inter­molecular O—H⋯O hydrogen bonds, which link the mol­ecules into centrosymmetric dimers. These dimers are further packed into stacks along the *b* axis by a slipped π–π inter­action between the furan and benzene rings of neighbouring mol­ecules [centroid–centroid distance = 3.727 (2) Å, inter­planar distance = 3.465 (2) Å and slippage = 1.373 (2) Å]. The crystal structure also exhibits a short S⋯O contact [S⋯O = 3.219 (2) Å].

## Related literature

For the pharmacological activity of benzofuran compounds, see: Aslam *et al.* (2009 ▶); Galal *et al.* (2009 ▶); Khan *et al.* (2005 ▶). For natural products with benzofuran rings, see: Akgul & Anil (2003 ▶); Soekamto *et al.* (2003 ▶). For the crystal structures of related compounds, see: Choi *et al.* (2009**a* ▶,b* ▶). 
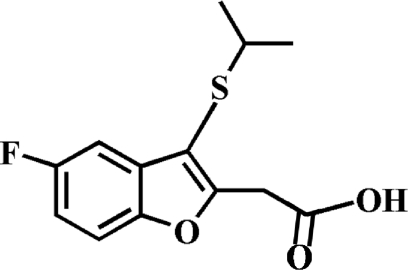


## Experimental

### 

#### Crystal data

C_13_H_13_FO_3_S
*M* *_r_* = 268.29
Monoclinic, 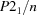

*a* = 10.6101 (2) Å
*b* = 8.5749 (1) Å
*c* = 13.6083 (2) Å
β = 97.149 (1)°
*V* = 1228.47 (3) Å^3^
*Z* = 4
Mo *K*α radiation
μ = 0.27 mm^−1^
*T* = 296 K
0.34 × 0.26 × 0.16 mm

#### Data collection

Bruker SMART APEXII CCD diffractometer
Absorption correction: multi-scan (*SADABS*; Bruker, 2009 ▶) *T*_min_ = 0.913, *T*_max_ = 0.958
11299 measured reflections
2812 independent reflections
2535 reflections with *I* > 2σ(*I*)
*R*_int_ = 0.026

#### Refinement

*R*[*F* ^2^ > 2σ(*F* ^2^)] = 0.032
*wR*(*F* ^2^) = 0.084
*S* = 1.08
2812 reflections
169 parameters
H atoms treated by a mixture of independent and constrained refinement
Δρ_max_ = 0.27 e Å^−3^
Δρ_min_ = −0.27 e Å^−3^

### 

Data collection: *APEX2* (Bruker, 2009 ▶); cell refinement: *SAINT* (Bruker, 2009 ▶); data reduction: *SAINT*; program(s) used to solve structure: *SHELXS97* (Sheldrick, 2008 ▶); program(s) used to refine structure: *SHELXL97* (Sheldrick, 2008 ▶); molecular graphics: *ORTEP-3* (Farrugia, 1997 ▶) and *DIAMOND* (Brandenburg, 1998 ▶); software used to prepare material for publication: *SHELXL97*.

## Supplementary Material

Crystal structure: contains datablock(s) global, I. DOI: 10.1107/S1600536811044436/vm2132sup1.cif

Structure factors: contains datablock(s) I. DOI: 10.1107/S1600536811044436/vm2132Isup2.hkl

Supplementary material file. DOI: 10.1107/S1600536811044436/vm2132Isup3.cml

Additional supplementary materials:  crystallographic information; 3D view; checkCIF report

## Figures and Tables

**Table 1:** Hydrogen-bond geometry (Å, °)

*D*—H⋯*A*	*D*—H	H⋯*A*	*D*⋯*A*	*D*—H⋯*A*
O2—H2⋯O3^i^	0.84 (2)	1.79 (2)	2.6257 (14)	177 (2)

Symmetry code: (i) 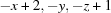.

**Fig. 1.**
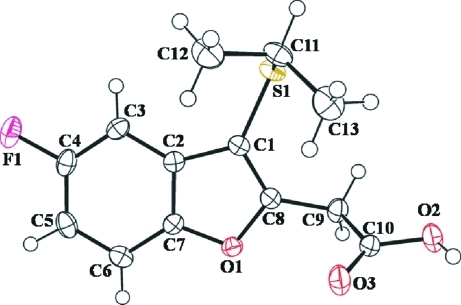
The molecular structure of the title compound with the atom numbering scheme. Displacement ellipsoids are drawn at the 50% probability level. H atoms are presented as small spheres of arbitrary radius.

**Fig. 2.**
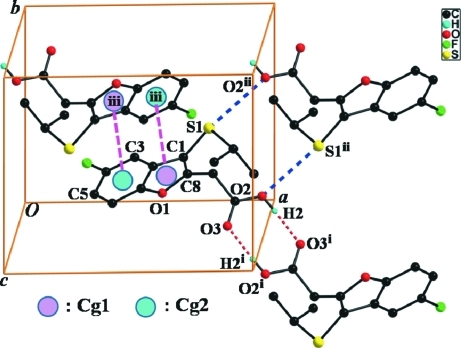
A view of the O—H···O, π–π and S···O interactions (dotted lines) in the crystal structure of the title compound. H atoms non-participating in hydrogen-bonding were omitted for clarity. [Symmetry codes: (i) - x + 2, - y, - z + 1 (ii) - x + 2 , - y + 1, - z + 1 (iii) - x + 1 , - y + 1, - z + 1]

## supplementary crystallographic information

### Comment

Substituted benzofuran derivatives have attracted considerable interest in view of their valuable pharmacological properties such as antibacterial and antifungal, antitumor and antiviral, and antimicrobial activities (Aslam *et al.*, 2009, Galal *et al.*, 2009, Khan *et al.*, 2005). These benzofuran derivatives occur in a wide range of natural products (Akgul & Anil, 2003; Soekamto *et al.*, 2003). As a part of our ongoing study of 2-(3-alkylsulfanyl-5-fluoro-1-benzofuran-2-yl)acetic acid analogues (Choi *et al.*, 2009**a*,b*), we report herein the crystal structure of the title compound.

In the title molecule (Fig. 1), the benzofuran unit is essentially planar, with a mean deviation of 0.014 (2) Å from the least-squares plane defined by the nine constituent atoms. In the crystal structure, the carboxyl groups are involved in intermolecular O—H···O hydrogen bonds (Table 1 & Fig. 2), which link the molecules into centrosymmetric dimers. These dimers are further packed into stacks along the *b* axis by a weak slipped π–π interaction between the furan and benzene rings of neighbouring molecules, with a Cg1···Cg2^iii^ distance of 3.727 (2) Å and an interplanar distance of 3.465 (2) Å resulting in a slippage of 1.373 (2) Å (Cg1 and Cg2 are the centroids of the C1/C2/C7/O1/C8 furan ring and the C2–C7 benzene ring, respectively). The crystal packing (Fig. 2) is further stabilized by an intermolecular S···O contact between the S atom and the O atom of the hydroxyl group [S1···O2^ii^ = 3.219 (2) Å].

### Experimental

Ethyl 2-(5-fluoro-3-isopropylsulfanyl-1-benzofuran-2-yl)acetate (355 mg, 1.2 mmol) was added to a solution of potassium hydroxide (337 mg. 6 mmol) in water (10 ml) and methanol (10 ml), and the mixture was refluxed for 5 h, then cooled. Water (10 ml) was added, and the solution was extracted with dichloromethane. The aqueous layer was acidified to pH 1 with concentrated hydrochloric acid and then extracted with chloroform, dried over magnesium sulfate, filtered and concentrated at reduced pressure. The residue was purified by column chromatography (ethyl acetate) to afford the title compound as a colorless solid [yield 89%, m.p. 399–400 K; *R*_f_ = 0.58 (ethyl acetate)]. Single crystals suitable for X-ray diffraction were prepared by slow evaporation of a solution of the title compound in diisopropyl ether at room temperature.

### Refinement

Atom H2 of the hydroxy group was found in a different Fourier map and refined freely. The other H atoms were positioned geometrically and refined using a riding model, with C—H = 0.93 Å for the aryl, 0.98 Å for the methine, 0.97 Å for the methylene, and 0.96 Å for the methyl H atoms. *U*_iso_(H) =1.2*U*_eq_(C) for the aryl, methine, and methylene H atoms, and 1.5*U*_eq_(C) for the methyl H atoms.

### Crystal data

**Table:**

C_13_H_13_FO_3_S	*F*(000) = 560
*M**_r_* = 268.29	*D*_x_ = 1.451 Mg m^−^^3^
Monoclinic, *P*2_1_/*n*	Mo *K*α radiation, λ = 0.71073 Å
Hall symbol: -P 2yn	Cell parameters from 6368 reflections
*a* = 10.6101 (2) Å	θ = 2.3–27.5°
*b* = 8.5749 (1) Å	µ = 0.27 mm^−^^1^
*c* = 13.6083 (2) Å	*T* = 296 K
β = 97.149 (1)°	Block, colourless
*V* = 1228.47 (3) Å^3^	0.34 × 0.26 × 0.16 mm
*Z* = 4	

### Data collection

**Table:**

Bruker SMART APEXII CCD diffractometer	2812 independent reflections
Radiation source: rotating anode	2535 reflections with *I* > 2σ(*I*)
graphite multilayer	*R*_int_ = 0.026
Detector resolution: 10.0 pixels mm^-1^	θ_max_ = 27.5°, θ_min_ = 2.3°
φ and ω scans	*h* = −9→13
Absorption correction: multi-scan (*SADABS*; Bruker, 2009)	*k* = −9→11
*T*_min_ = 0.913, *T*_max_ = 0.958	*l* = −17→17
11299 measured reflections	

### Refinement

**Table:**

Refinement on *F*^2^	Primary atom site location: structure-invariant direct methods
Least-squares matrix: full	Secondary atom site location: difference Fourier map
*R*[*F*^2^ > 2σ(*F*^2^)] = 0.032	Hydrogen site location: difference Fourier map
*wR*(*F*^2^) = 0.084	H atoms treated by a mixture of independent and constrained refinement
*S* = 1.08	*w* = 1/[σ^2^(*F*_o_^2^) + (0.0391*P*)^2^ + 0.4795*P*] where *P* = (*F*_o_^2^ + 2*F*_c_^2^)/3
2812 reflections	(Δ/σ)_max_ < 0.001
169 parameters	Δρ_max_ = 0.27 e Å^−^^3^
0 restraints	Δρ_min_ = −0.27 e Å^−^^3^

### Special details

**Table:**

Geometry. All esds (except the esd in the dihedral angle between two l.s. planes) are estimated using the full covariance matrix. The cell esds are taken into account individually in the estimation of esds in distances, angles and torsion angles; correlations between esds in cell parameters are only used when they are defined by crystal symmetry. An approximate (isotropic) treatment of cell esds is used for estimating esds involving l.s. planes.
Refinement. Refinement of F^2^ against ALL reflections. The weighted R-factor wR and goodness of fit S are based on F^2^, conventional R-factors R are based on F, with F set to zero for negative F^2^. The threshold expression of F^2^ > 2sigma(F^2^) is used only for calculating R-factors(gt) etc. and is not relevant to the choice of reflections for refinement. R-factors based on F^2^ are statistically about twice as large as those based on F, and R- factors based on ALL data will be even larger.

### Fractional atomic coordinates and isotropic or equivalent isotropic displacement parameters (Å^2^)

**Table:**

	*x*	*y*	*z*	*U*_iso_*/*U*_eq_	
S1	0.78862 (3)	0.58282 (4)	0.59632 (2)	0.02413 (11)	
F1	0.31233 (8)	0.47027 (11)	0.75555 (6)	0.0345 (2)	
O1	0.59098 (8)	0.21589 (10)	0.48893 (6)	0.0214 (2)	
O2	0.98666 (9)	0.17121 (12)	0.42302 (8)	0.0282 (2)	
H2	1.039 (2)	0.102 (3)	0.4435 (17)	0.061 (7)*	
O3	0.85509 (9)	0.05406 (12)	0.51590 (8)	0.0315 (2)	
C1	0.68384 (12)	0.42599 (14)	0.56964 (9)	0.0193 (3)	
C2	0.56451 (12)	0.39600 (14)	0.60737 (9)	0.0193 (3)	
C3	0.49739 (12)	0.46787 (15)	0.67707 (9)	0.0231 (3)	
H3	0.5284	0.5554	0.7126	0.028*	
C4	0.38297 (13)	0.40136 (16)	0.68987 (10)	0.0250 (3)	
C5	0.33252 (12)	0.26984 (17)	0.64039 (10)	0.0263 (3)	
H5	0.2549	0.2300	0.6537	0.032*	
C6	0.39816 (12)	0.19808 (16)	0.57121 (10)	0.0241 (3)	
H6	0.3672	0.1096	0.5368	0.029*	
C7	0.51263 (12)	0.26584 (15)	0.55626 (9)	0.0194 (3)	
C8	0.69308 (11)	0.31625 (14)	0.49921 (9)	0.0198 (3)	
C9	0.79023 (13)	0.29168 (16)	0.43123 (10)	0.0245 (3)	
H9A	0.8387	0.3871	0.4280	0.029*	
H9B	0.7475	0.2707	0.3653	0.029*	
C10	0.88063 (12)	0.15988 (15)	0.46143 (9)	0.0212 (3)	
C11	0.89540 (13)	0.51469 (17)	0.70409 (10)	0.0266 (3)	
H11	0.9545	0.6004	0.7233	0.032*	
C12	0.82624 (15)	0.4816 (2)	0.79269 (11)	0.0363 (4)	
H12A	0.7690	0.3957	0.7779	0.055*	
H12B	0.7791	0.5723	0.8076	0.055*	
H12C	0.8868	0.4558	0.8488	0.055*	
C13	0.97563 (14)	0.3767 (2)	0.67876 (12)	0.0370 (4)	
H13A	1.0382	0.3538	0.7341	0.056*	
H13B	1.0173	0.4019	0.6221	0.056*	
H13C	0.9220	0.2874	0.6642	0.056*	

### Atomic displacement parameters (Å^2^)

**Table:**

	*U*^11^	*U*^22^	*U*^33^	*U*^12^	*U*^13^	*U*^23^
S1	0.02556 (18)	0.01943 (17)	0.02593 (18)	−0.00434 (12)	−0.00267 (13)	0.00363 (12)
F1	0.0348 (5)	0.0401 (5)	0.0317 (4)	0.0130 (4)	0.0158 (4)	0.0031 (4)
O1	0.0213 (4)	0.0201 (4)	0.0227 (4)	−0.0003 (4)	0.0024 (3)	−0.0032 (3)
O2	0.0196 (5)	0.0303 (5)	0.0363 (5)	0.0019 (4)	0.0095 (4)	0.0084 (4)
O3	0.0273 (5)	0.0289 (5)	0.0413 (6)	0.0064 (4)	0.0158 (4)	0.0119 (4)
C1	0.0185 (6)	0.0180 (6)	0.0207 (6)	−0.0002 (5)	−0.0002 (5)	0.0021 (5)
C2	0.0188 (6)	0.0187 (6)	0.0197 (6)	0.0029 (5)	−0.0001 (5)	0.0020 (5)
C3	0.0270 (7)	0.0205 (6)	0.0216 (6)	0.0047 (5)	0.0026 (5)	−0.0008 (5)
C4	0.0253 (7)	0.0287 (7)	0.0219 (6)	0.0103 (5)	0.0069 (5)	0.0054 (5)
C5	0.0193 (6)	0.0305 (7)	0.0292 (7)	0.0013 (5)	0.0035 (5)	0.0101 (6)
C6	0.0217 (6)	0.0228 (6)	0.0266 (6)	−0.0014 (5)	−0.0018 (5)	0.0027 (5)
C7	0.0193 (6)	0.0196 (6)	0.0191 (5)	0.0037 (5)	0.0007 (4)	0.0013 (5)
C8	0.0182 (6)	0.0194 (6)	0.0212 (6)	0.0013 (5)	0.0004 (5)	0.0024 (5)
C9	0.0253 (7)	0.0255 (7)	0.0237 (6)	0.0034 (5)	0.0067 (5)	0.0029 (5)
C10	0.0209 (6)	0.0224 (6)	0.0209 (6)	−0.0013 (5)	0.0051 (5)	−0.0022 (5)
C11	0.0249 (7)	0.0266 (7)	0.0261 (7)	−0.0072 (5)	−0.0059 (5)	0.0037 (5)
C12	0.0394 (8)	0.0435 (9)	0.0245 (7)	−0.0098 (7)	−0.0023 (6)	0.0027 (6)
C13	0.0276 (7)	0.0404 (9)	0.0410 (8)	0.0041 (7)	−0.0040 (6)	0.0072 (7)

### Geometric parameters (Å, °)

**Table:**

S1—C1	1.7539 (13)	C5—H5	0.9300
S1—C11	1.8335 (13)	C6—C7	1.3840 (18)
S1—O2^i^	3.2185 (10)	C6—H6	0.9300
F1—C4	1.3699 (15)	C8—C9	1.4832 (17)
O1—C8	1.3769 (15)	C9—C10	1.5060 (18)
O1—C7	1.3797 (15)	C9—H9A	0.9700
O2—C10	1.3018 (15)	C9—H9B	0.9700
O2—H2	0.84 (2)	C11—C12	1.514 (2)
O3—C10	1.2232 (16)	C11—C13	1.522 (2)
C1—C8	1.3553 (18)	C11—H11	0.9800
C1—C2	1.4472 (17)	C12—H12A	0.9600
C2—C7	1.3919 (18)	C12—H12B	0.9600
C2—C3	1.3981 (17)	C12—H12C	0.9600
C3—C4	1.3719 (19)	C13—H13A	0.9600
C3—H3	0.9300	C13—H13B	0.9600
C4—C5	1.386 (2)	C13—H13C	0.9600
C5—C6	1.3832 (19)		
			
C1—S1—C11	103.53 (6)	O1—C8—C9	116.54 (11)
C1—S1—O2^i^	160.48 (5)	C8—C9—C10	113.88 (11)
C11—S1—O2^i^	83.33 (5)	C8—C9—H9A	108.8
C8—O1—C7	105.66 (9)	C10—C9—H9A	108.8
C10—O2—H2	112.4 (15)	C8—C9—H9B	108.8
C8—C1—C2	105.73 (11)	C10—C9—H9B	108.8
C8—C1—S1	125.38 (10)	H9A—C9—H9B	107.7
C2—C1—S1	128.52 (10)	O3—C10—O2	124.50 (12)
C7—C2—C3	119.30 (12)	O3—C10—C9	122.72 (11)
C7—C2—C1	105.91 (11)	O2—C10—C9	112.79 (11)
C3—C2—C1	134.78 (12)	C12—C11—C13	112.00 (13)
C4—C3—C2	115.87 (12)	C12—C11—S1	112.60 (10)
C4—C3—H3	122.1	C13—C11—S1	111.96 (10)
C2—C3—H3	122.1	C12—C11—H11	106.6
F1—C4—C3	117.92 (13)	C13—C11—H11	106.6
F1—C4—C5	117.27 (12)	S1—C11—H11	106.6
C3—C4—C5	124.81 (12)	C11—C12—H12A	109.5
C6—C5—C4	119.72 (12)	C11—C12—H12B	109.5
C6—C5—H5	120.1	H12A—C12—H12B	109.5
C4—C5—H5	120.1	C11—C12—H12C	109.5
C5—C6—C7	116.02 (12)	H12A—C12—H12C	109.5
C5—C6—H6	122.0	H12B—C12—H12C	109.5
C7—C6—H6	122.0	C11—C13—H13A	109.5
O1—C7—C6	125.42 (12)	C11—C13—H13B	109.5
O1—C7—C2	110.32 (11)	H13A—C13—H13B	109.5
C6—C7—C2	124.26 (12)	C11—C13—H13C	109.5
C1—C8—O1	112.37 (11)	H13A—C13—H13C	109.5
C1—C8—C9	131.05 (12)	H13B—C13—H13C	109.5
			
C11—S1—C1—C8	−98.49 (12)	C3—C2—C7—O1	177.94 (11)
O2^i^—S1—C1—C8	10.3 (2)	C1—C2—C7—O1	−0.95 (13)
C11—S1—C1—C2	89.60 (12)	C3—C2—C7—C6	−1.66 (19)
O2^i^—S1—C1—C2	−161.66 (10)	C1—C2—C7—C6	179.46 (12)
C8—C1—C2—C7	1.13 (13)	C2—C1—C8—O1	−0.95 (14)
S1—C1—C2—C7	174.29 (10)	S1—C1—C8—O1	−174.38 (9)
C8—C1—C2—C3	−177.50 (14)	C2—C1—C8—C9	176.54 (13)
S1—C1—C2—C3	−4.3 (2)	S1—C1—C8—C9	3.1 (2)
C7—C2—C3—C4	0.35 (18)	C7—O1—C8—C1	0.38 (13)
C1—C2—C3—C4	178.84 (13)	C7—O1—C8—C9	−177.51 (10)
C2—C3—C4—F1	−178.33 (11)	C1—C8—C9—C10	103.58 (16)
C2—C3—C4—C5	1.0 (2)	O1—C8—C9—C10	−79.02 (14)
F1—C4—C5—C6	178.22 (11)	C8—C9—C10—O3	21.68 (19)
C3—C4—C5—C6	−1.1 (2)	C8—C9—C10—O2	−158.70 (11)
C4—C5—C6—C7	−0.16 (18)	C1—S1—C11—C12	−61.64 (12)
C8—O1—C7—C6	179.98 (12)	O2^i^—S1—C11—C12	136.93 (11)
C8—O1—C7—C2	0.39 (13)	C1—S1—C11—C13	65.60 (11)
C5—C6—C7—O1	−178.00 (11)	O2^i^—S1—C11—C13	−95.82 (10)
C5—C6—C7—C2	1.53 (19)		

Symmetry codes: (i) −*x*+2, −*y*+1, −*z*+1.

### Hydrogen-bond geometry (Å, °)

**Table:**

*D*—H···*A*	*D*—H	H···*A*	*D*···*A*	*D*—H···*A*
O2—H2···O3^ii^	0.84 (2)	1.79 (2)	2.6257 (14)	177 (2)

Symmetry codes: (ii) −*x*+2, −*y*, −*z*+1.
